# Antioxidant and anti-inflammatory potential of spirulina and thymoquinone mitigate the methotrexate-induced neurotoxicity

**DOI:** 10.1007/s00210-023-02739-4

**Published:** 2023-09-29

**Authors:** Alaa Behairy, Ashraf Elkomy, Faten Elsayed, Mohamed M. S. Gaballa, Ahmed Soliman, Mohamed Aboubakr

**Affiliations:** 1 Department of Pharmacology, Faculty of Veterinary Medicine, Benha University, Moshtohor, Toukh, 13736 Qaliobiya Egypt; 2 Department of Pathology, Faculty of Veterinary Medicine, Benha University, Moshtohor, Toukh, 13736 Qaliobiya Egypt; 3 Department of Pharmacology, Faculty of Veterinary Medicine, Cairo University, Giza, 12211 Egypt

**Keywords:** Antioxidants, Methotrexate, Brain, Inflammation, Spirulina, Thymoquinone

## Abstract

The objective of this study was to investigate whether the neurotoxic effects caused by methotrexate (MTX), a frequently used chemotherapy drug, could be improved by administering *Spirulina platensis* (SP) and/or thymoquinone (TQ). Seven groups of seven rats were assigned randomly for duration of 21 days. The groups consisted of a control group that was given saline only. The second group was given 500 mg/kg of SP orally; the third group was given 10 mg/kg of TQ orally. The fourth group was given a single IP dose of 20 mg/kg of MTX on the 15^th^ day of the experiment. The fifth group was given both SP and MTX, the sixth group was given both TQ and MTX, and the seventh group was given SP, TQ, and MTX. After MTX exposure, the study found that AChE inhibition, depletion of glutathione, and increased levels of MDA occurred. MTX also decreased the activity of SOD and CAT, as well as the levels of inflammatory mediators such as IL-1, IL-6, and tumor necrosis factor-α. MTX induced apoptosis in brain tissue. However, when MTX was combined with either SP or TQ, the harmful effects on the body were significantly reduced. This combination treatment resulted in a faster return to normal levels of biochemical, oxidative markers, inflammatory responses, and cell death. In conclusion, supplementation with SP or TQ could potentially alleviate MTX-induced neuronal injury, likely due to their antioxidant, anti-inflammatory, and anti-apoptotic effects.

## Introduction

Methotrexate (MTX) belongs to the antimetabolite group of anti-cancer drugs and has the ability to easily pass through the blood–brain barrier (Suwannakot et al. 2022; Aboubakr et al. 2023a). By binding to and inhibiting the enzyme dihydrofolate reductase, the process of dihydrofolate conversion to tetrahydrofolate is halted by MTX, leading to the inhibition of DNA synthesis (Vazi et al. 2021). MTX is frequently prescribed for treating different cancer types such as acute lymphoblastic leukemia and lymphoma, as well as inflammatory conditions like rheumatoid arthritis (Ahmed et al. 2021; Aboubakr et al. 2023c). The administration of this medication may result in a range of adverse effects, including cardiological issues like arrhythmia, gastrointestinal symptoms such as nausea and vomiting, and psychological disorders like depression. Additionally, the drug can lead to immunosuppression (Yang et al. 2011).

In normal physiological circumstances, cells maintain a balance between the production of reactive oxygen species (ROS) and antioxidant enzyme activity (Dasuri et al. 2013). However, if there is a reduction in the number of antioxidant enzymes, this balance is disruptd, resulting in ROS-induced damage to the cell (Sallam et al. 2021; Elsayed et al. 2022). MTX causes an increase in ROS formation, ultimately leading to cell cycle arrest and death in the central nervous system (Naewla et al. 2019; Aslankoc et al. 2022). Several recent studies have shown that the use of MTX in treatment can lead to severe neurotoxicity, a reduction in hippocampal neurogenesis, and changes in memory function (Naewla et al. 2019; Sirichoat et al. 2019; Sritawan et al. 2020; Welbat et al. 2020; Ahmed et al. 2021; Aslankoc et al. 2022). MTX-induced oxidative stress can trigger inflammatory responses by increasing cytokine levels (Rashid et al. 2013; Kandemir et al. 2017). The brain is especially vulnerable to oxidative damage because of its low antioxidant defenses, high energy demands, and the presence of polyunsaturated fatty acids (Soni et al. 2018). Furthermore, (Shagirtha et al. 2017) suggest that the restricted ability of neurons to synthesize glutathione could exacerbate the neurotoxic effects of MTX caused by oxidative stress. Previous research has indicated that memory impairments in chemotherapy patients are often linked to hippocampal dysfunction (Yang et al. 2012, 2014). However, protecting against MTX toxicity in the brain can be achieved by reducing oxidative stress.

*Spirulina platensis* (SP) is a filamentous cyanobacterium that is enriched with nutrients and has various medical uses (Abdel-Daim et al. 2015). SP includes natural antioxidants and free radical scavengers, including phenolic compounds, tocopherol, γ-linolenic acid, β-carotene, and phycocyanin (Khan et al. 2005). Moreover, both SP and its primary component, C-phycocyanin, possess immunomodulatory, anti-inflammatory, neuroprotective properties, and anticancer (Reddy et al. 2000; Romay et al. 2003). SP has become increasingly popular due to its safety profile and remarkable neuroprotective properties against toxicity caused by various chemicals and pollutants, such as lead (Khalil et al. 2018; Galal et al. 2019), manganese (Ibrahim et al. 2020), acrylamide (Bin-Jumah et al. 2021), and microcystin-LR (Germoush et al. 2022).

Thymoquinone (TQ) is a chemical compound derived from the seeds of Nigella sativa. Previous research on TQ has demonstrated its pharmacological properties, including anti-inflammatory, anti-tumor, neuroprotective, and antioxidant effects (Ashraf et al. 2011; Abdel-Daim et al. 2020). TQ has been reported to have antioxidant effects by scavenging free radicals and enhancing the activity of internal antioxidant enzymes (Badary et al. 2003), which has been associated with its neuroprotective activity (Saygin et al. 2018; Abdel-Daim et al. 2019, 2020; Aboubakr et al. 2021; Kaymak et al. 2021).

This study aims to explore the potential protective effects of SP and/or TQ against MTX-induced neurotoxicity in rats, taking into account the previously reported beneficial pharmacological properties of these compounds.

## Materials and methods

### Chemicals

The Methotrexate injectable solution with a concentration of 50 mg/5 mL was obtained from Mina Pharm Pharmaceuticals located in Cairo, Egypt. Thymoquinone with 98% purity was supplied by Sigma Aldrich (Saint Louis, MO, USA). Spirulina platensis was obtained from the Algal Biotechnology unit at the National Research Center located in Dokki, Cairo, Egypt. Biodiagnostic CO, Cairo, Egypt provided kits for assessing the levels of MDA, SOD, GSH, and CAT. R&D, Mannheim, Germany provided AChE assay kits. ELISA kits to evaluate cytokines IL-1β, IL-6, and TNF-α (Mybiosource Company, USA) to measure inflammatory processes.

### Animals and experimental design

A total of 49 male Wister Albino rats, weighing between 188–222 g, were procured from the Egyptian Organization for Biological Products and Vaccines. The rats were housed in a regulated environment at a temperature of 25 ± 2°C, with a 12-h light/dark cycle, and were provided with a standard pellet diet and unrestricted access to water. The rats were allowed to acclimate for one week before the study began, following which they were divided into seven groups, each comprising seven rats. The control group received only saline, while the second group received SP (500 mg/kg/day po for 21 days) (Khafaga and El-Sayed 2018). The third group was administered TQ (10 mg/kg/day po for 21 days) (Abdel-Daim et al. 2020). The fourth group was given saline, PO, and a single dose of MTX (20 mg/kg, IP) on the 15^th^ day of the study, which served as a toxic control for MTX (Khafaga and El-Sayed 2018). The fifth group was given SP + MTX, the sixth group received TQ + MTX, and the seventh group was administered SP + TQ + MTX.

### Blood sampling and serum biochemical markers

Following the completion of the experiment, the rats were given isoflurane anesthesia, and blood samples were collected from the retro-orbital plexus. These samples were centrifuged for 15 min at 1200 g, and the serum obtained was preserved at a temperature of -20°C to facilitate future biochemical analysis. The activity of serum AChE was quantified using the method delineated by (Ellman et al. 1961). The levels of cytokines (IL-1, IL-6, and TNF-α) were evaluated by ELISA kits as per the manufacturer's guidelines.

### Tissue sampling and oxidative stress markers

To create a brain homogenate, 10% of brain tissue was combined with an ice-cold saline solution, and the resultant mixture was centrifuged at 2500 rpm for 10 min at 4°C. Antioxidant markers such as GSH, SOD, MDA, and CAT were assessed from the resulting mixture. The methods employed to determine these markers were as follows: GSH (Beutler et al. 1963), SOD (Nishikimi et al. 1972), MDA (Ohkawa et al. 1979), and CAT (Cohen et al. 1970).

### Histopathological examination

The brain of each animal was extracted with caution, immersed in a 10% formalin solution, and dehydrated gradually using increasing concentrations of alcohol. Next, the brain was cleared in xylene and embedded in paraffin. Sections of 5 μm thickness were produced from the paraffin block and subjected to hematoxylin and eosin (H&E) staining (Bancroft et al. 2013).

### Histopathological scoring of brain injury severity

To ascertain the histological alterations in the brain, a structured numerical scoring system was employed:

- Score (-): Indicates unaltered, normal histology.
- Score ( +): Indicates the presence of mild histological alterations.
- Score (+ +): Indicates moderate histological alterations.
- Score (+ + +): Indicates severe histological alterations.

Utilizing a 40 × objective, a thorough examination of various areas within each section was undertaken. This rigorous evaluation aimed to evaluate the severity of the following specific neuronal morphological changes: Degenerated Shrinkage of neuronal cell body, nuclear pyknosis, dense eosinophilic cytoplasm, chromatolysis, degeneration of Purkinje cells leads to a shrunken appearance, notable loss of Purkinje cells, diminished thickness of the pyramidal cell layer, and neuronal disarray.

Each parameter was systematically scored, leading to an understanding of the overall injury severity degree. The adopted methodology for scoring was based on a modified system, as explained (Li et al. 2013; Yang et al. 2019).

### Immunohistochemical staining

In order to detect Bax, a protein associated with apoptosis, the researchers employed the streptavidin-peroxidase method (Hassanen et al. 2022). The brain sections were first deparaffinized and hydrated, followed by three washes with phosphate-buffered saline (PBS) and the use of 3% H_2_O_2_ to neutralize the natural peroxidase activity. After another rinse with PBS, a blocking buffer consisting of normal low-lethal serum was applied. The sections were then subjected to immunohistochemical staining. They were incubated overnight at 4 ◦C with a diluted solution (1:50) of rabbit polyclonal anti-Bax antibody obtained from Abcam Co, USA. Next, the sections were treated with a secondary antibody and avidin–biotin complex. The stain 3,3′-diaminobenzidine was utilized as a chromogen, and Mayer's hematoxylin was used to counterstain the sections. Finally, the sections were examined under a light microscope.

### Immunohistochemistry staining scoring

Our study evaluated the immunohistochemistry (IHC) staining using a scoring system consisting of four categories. A pathologist, blinded to the experimental groups, independently scored the sections. This scoring system assessed the number of immunoreactive cells and the intensity of immunoreactivity (IR) at the individual cell level. The resulting scores comprehensively evaluated the immunoreactive characteristics within each sample (Partoazar et al. 2021). The scoring categories were defined as follows:

- Negative (0–1): Indicates the absence of immunoreactive cells.
- Weak (2–3): Represents a small number of cells with intermediate immunoreactivity.
- Moderate (4–6): Represents a substantial number of cells with intermediate to strong immunoreactivity.
- Severe (7–8): Indicates a large number of cells with intense and extensive immunoreactivity.

### Statistical analysis

The results were expressed using mean ± SD. Statistical analysis was carried out using GraphPad Prism 9 software (San Diego, CA, USA) with one-way ANOVA and Tukey’s post hoc test for multiple comparisons. Statistical significance was determined by asterisks, with * indicating p < 0.05, ** indicating p < 0.01, *** indicating p < 0.001, and **** indicating p < 0.0001, compared to the MTX-treated groups.

## Results

### AChE activity

**Fig. 1:**
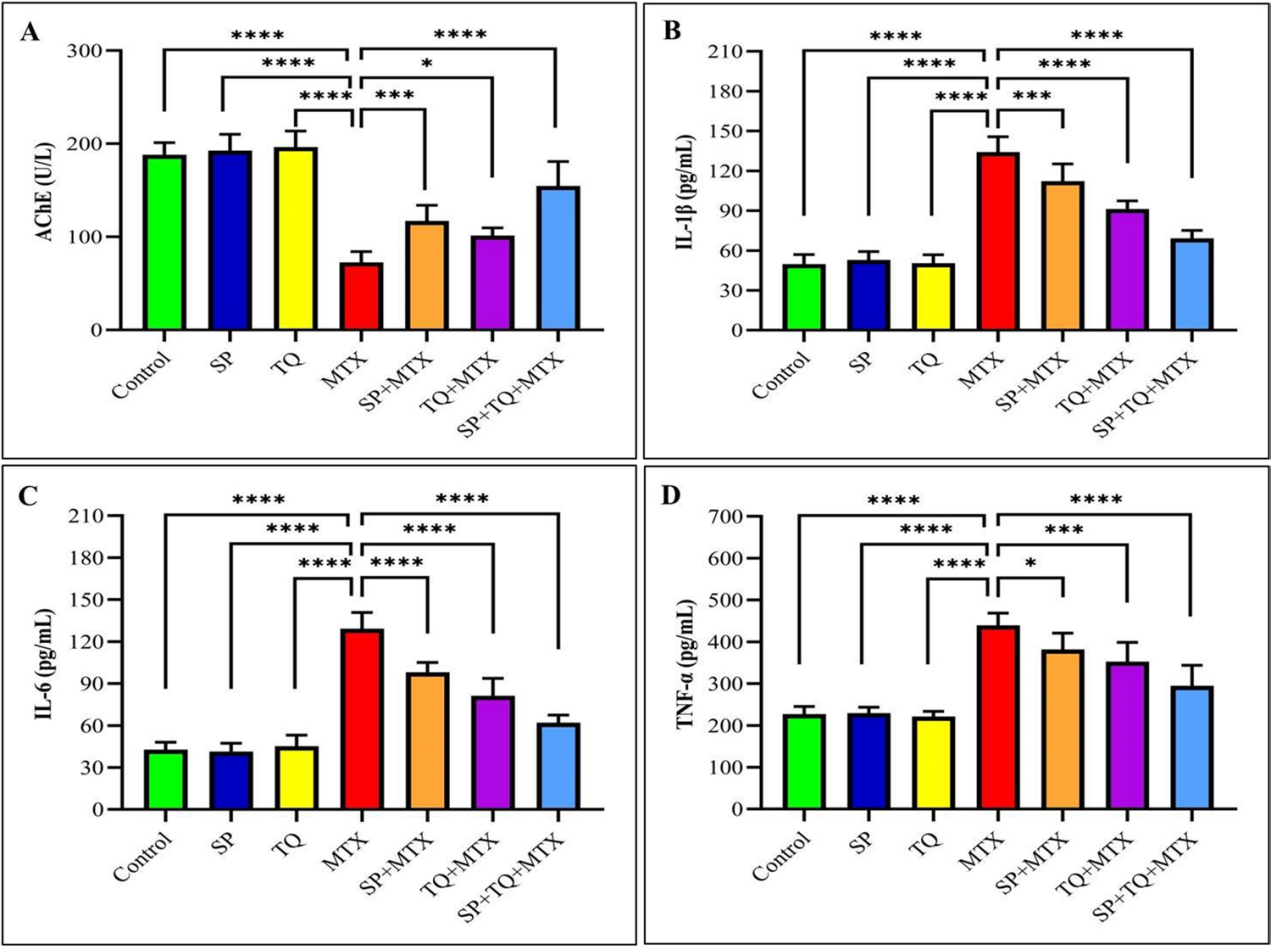
Effect of spirulina (SP) and/or thymoquinone (TQ) on serum AChE activity and IL-1β, IL-6, TNF-α levels against methotrexate (MTX) induced neurotoxicity in rats (*n*=7). **A** (AchE); **B** (IL-1β); **C** ((IL-6); **D** (TNF-α). Statistical significance was determined by asterisks, with * indicating *p* < 0.05, *** indicating *p* < 0.001, and **** indicating *p* < 0.0001, compared to the MTX-treated groups

This study aimed to explore whether SP or TQ could act as potent antioxidants to protect rat brains from MTX-induced neurotoxicity. The administration of MTX resulted in a marked decrease in AChE activity. However, the rats treated with either SP or TQ showed a slight elevation in AChE activity in their brain tissue, as illustrated in Fig. 1.

### Inflammatory cytokines markers

In order to evaluate the potential anti-inflammatory effects of SP and/or TQ on MTX-induced neurotoxicity, the levels of IL-1β, IL-6, and TNF-α in the brain tissue of rats were measured. The results of the study demonstrated that exposure to MTX caused a significant increase in the levels of TNF-α, IL-1β, and IL-6 in the brain tissue of rats, as shown in Fig. 1, in comparison to the control group. Nevertheless, the administration of SP and/or TQ in combination with MTX reduced the elevation of these markers, suggesting that they are effective in mitigating the inflammation induced by MTX neurotoxicity.

### Brain lipid peroxidation and antioxidant status

**Fig. 2:**
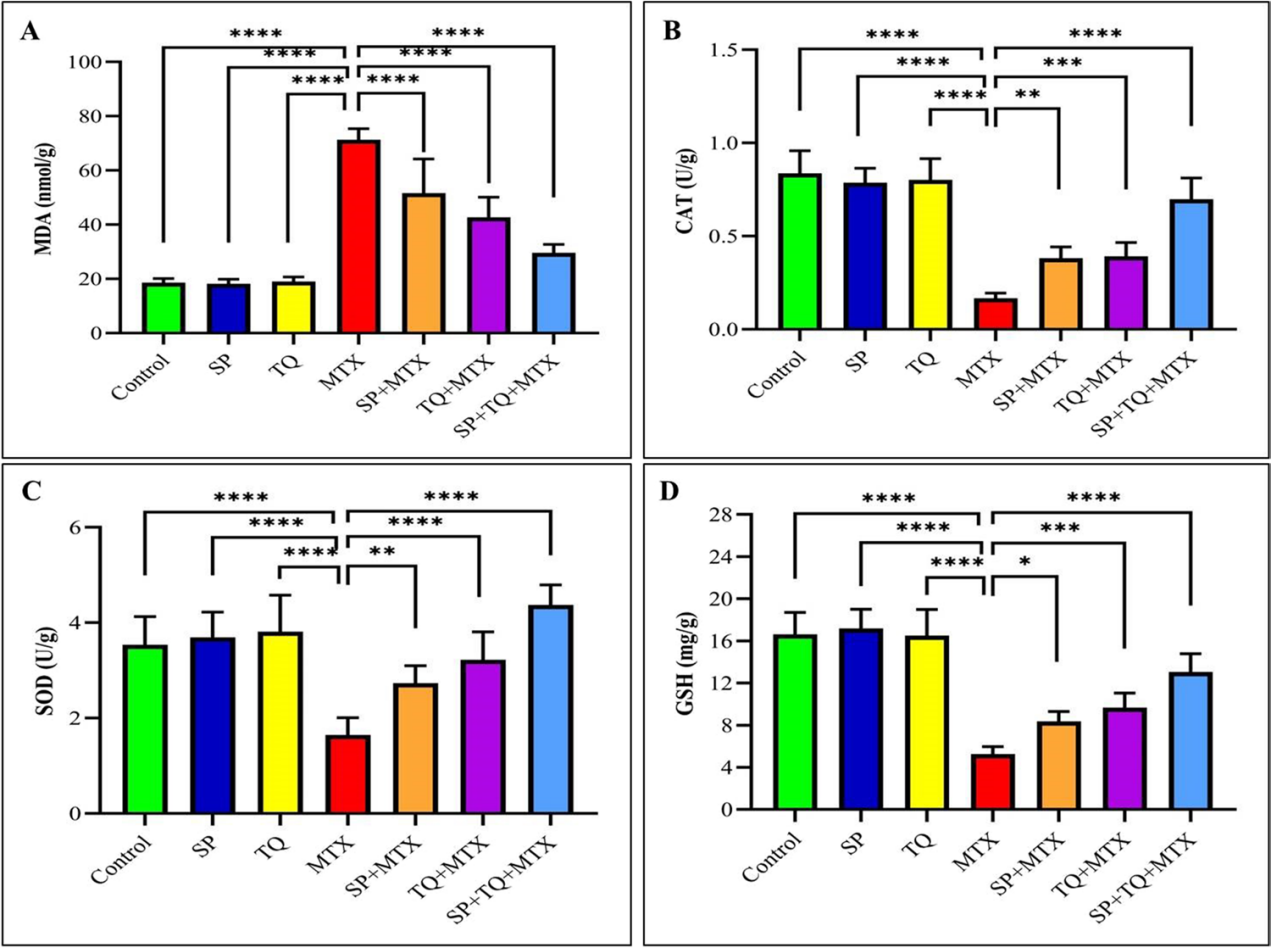
Effect of spirulina (SP), thymoquinone (TQ) and methotrexate (MTX) on antioxidant parameters in brain tissues in rats (*n*=7). **A** (MDA); **B** (CAT); **C** (SOD); **D** (GSH). Statistical significance was determined by asterisks, with * indicating *p* < 0.05, ** indicating *p* < 0.01, *** indicating *p* < 0.001, and **** indicating *p* < 0.0001, compared to the MTX-treated groups

According to the findings of the research, MTX caused oxidative stress, which was demonstrated by a marked increase in MDA levels and a significant decline in GSH content, as well as CAT and SOD activities, in comparison to the control group, as illustrated in Fig. 2.

### Histopathological findings

**Fig. 3:**
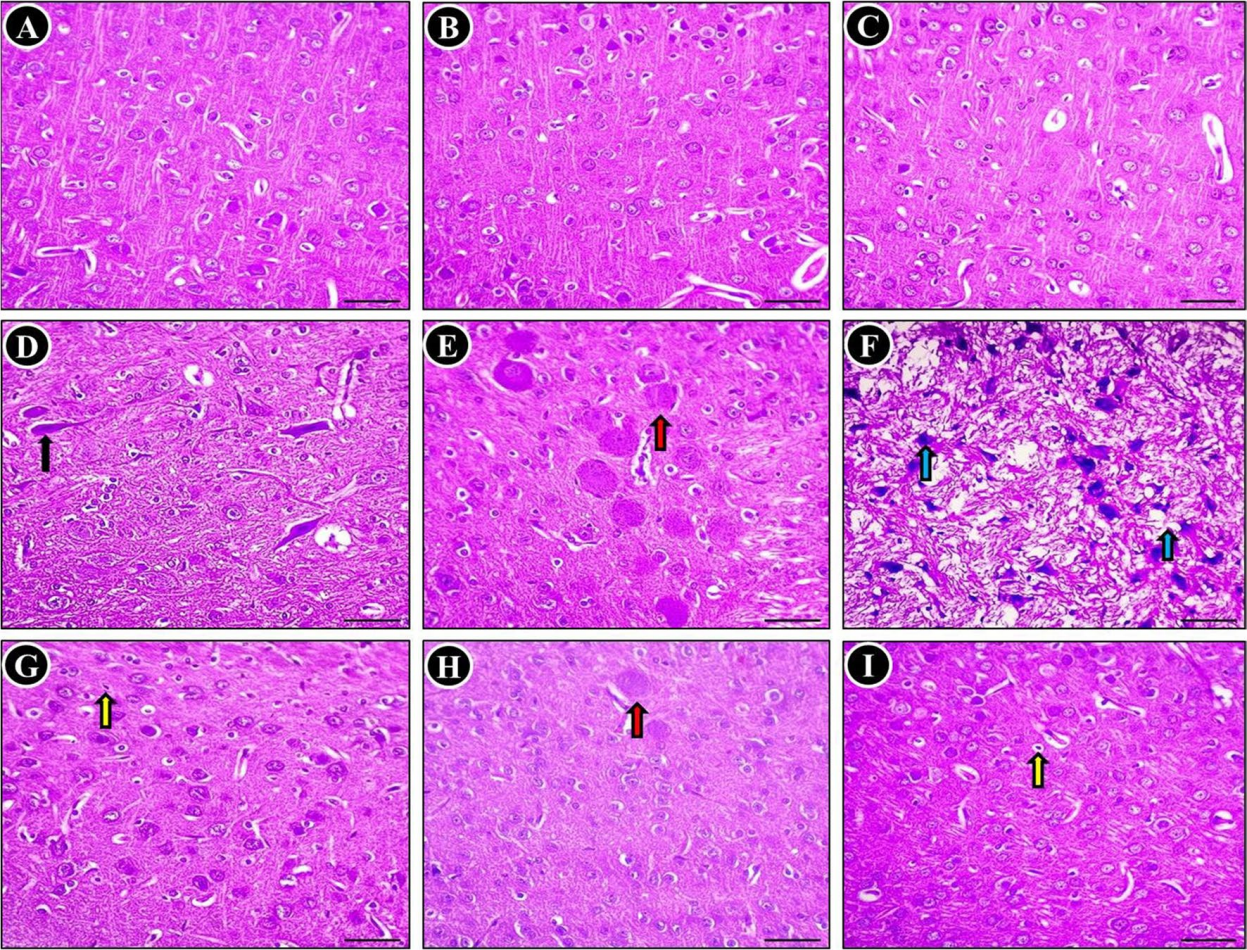
Histopathological examination of the cerebrum was conducted on different groups (H&E stain). The analysis revealed the presence of seemingly normal neurons in the control group (**A**), as well as in the SP-treated (**B**) and TQ-treated (**C**) groups. However, significant abnormalities were observed in the MTX-treated group, characterized by neurofibrillary tangles (indicated by a black arrow) (**D**), neuronal degeneration (indicated by a red arrow) (**E**), and neuropile vacuolation basophilic necrotic neuron (indicated by a blue arrow) (**F**). Nevertheless, a small number of shrunken apoptotic neurons (indicated by yellow arrow) were observed in the SP + MTX-treated (**G**), TQ + MTX-treated (**H**), and SP + TQ + MTX combination (**I**) groups

The histopathological alterations were examined in the cerebral cortex tissue subsequent to exposure to MTX, with the aim of highlighting the findings obtained. The histological architecture of the cerebral cortex tissue specimens from the control (Fig. 3A), SP-treated (Fig. 3B), and TQ-treated (Fig. 3C) groups exhibited normal neuroglial cells arranged in several layers. In contrast, the cerebral cortex following MTX intoxication showed various neurodegeneration. The degenerated neurons displayed neurofibrillary tangles, neuropile vaculation, indistinct cell boundaries, and some displayed chromatolysis as well as pyknotic nucleus (Fig. 3D-F). When MTX was administered concurrently with SP, a notable reduction in the number of degenerated and necrotic neurons was observed compared to untreated MTX rats (Fig. 3G). Furthermore, there was no evidence of vacuolation in the neuropil. Foci of degenerated/necrotic neurons, and pyknotic nucleus were still present in the cerebral cortex of MTX + TQ-treated rats (Fig. 3H). The neuroprotective effect was more prominent in the MTX + SP + TQ-treated group, as evidenced by significant improvement in the MTX-induced histopathological lesions. Only a few shrunken neurons were observed in the cerebral cortex of these groups (Fig. 3I).

**Fig. 4:**
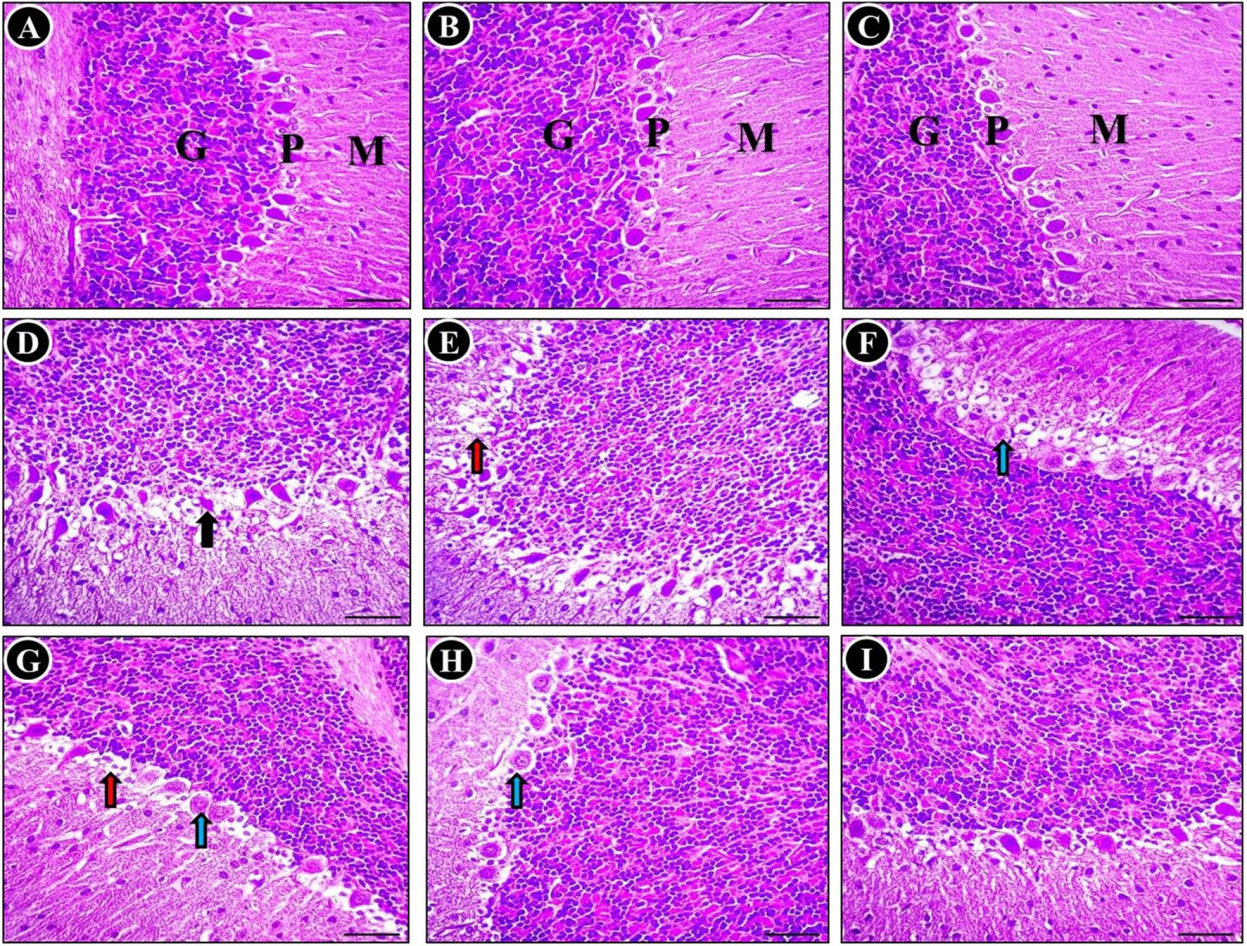
Histopathological examination of the cerebellum was conducted in various groups (H&E stain). The histological findings revealed nearly normal Purkinje neurons in the control group (**A**), as well as in the SP-treated (**B**) and TQ-treated (**C**) groups, with minimal degeneration observed in a small number of neurons. Molecular (M), Purkinje (P) and granular (G) layers in the control groups. In contrast, the cerebellum of rats treated with MTX exhibited significant aberrations, including Purkinje cell shrinkage (indicated by black arrows) (**D)**, loss of cells (indicated by red arrows) (**E**), and reduced presence of dendrites (indicated by blue arrows) (**F**). However, in the SP (**G**), TQ (**H**), and combination (**I**) groups co-treated with MTX, a notable reduction in degenerated/apoptotic Purkinje cells was observed

Regarding the histological examination of the cerebellum in different experimental groups, it was found that the control group, as shown in Fig. 4A, exhibited a typical histological representation of the normal gray matter. This gray matter was observed to be composed of three distinct layers: the molecular layer(M), the Purkinje layer (P), and the granular layer (G). Similarly, the SP-treated group (Fig. 4B) and TQ-treated group (Fig. 4C) also displayed normal brain tissue architecture. In contrast, rats intoxicated with MTX showed significant abnormalities, including shrunken, degenerated Purkinje cells with varying degrees of vaculation. Moreover, the predominant observations in this group, as depicted in were the loss of some Purkinje cells, and neuronophagia (Fig. 4D-F). However, when SP or TQ was administered concurrently with MTX intoxication, the cerebellar architecture showed a relatively restored and normal appearance compared to untreated MTX rats however displayed a reduced presence of dendrites in focal areas could be still seen. This can be seen in Fig. 4G (SP + MTX) and Fig. 4H (TQ + MTX), where a significant decrease in the severity and distribution of cerebellar lesions was observed. Notably, the number of necrotic Purkinje cells in the cerebellar cortex decreased, and restoration of the molecular cell layer was observed. Furthermore, in rats treated with SP + TQ + MTX), a remarkable improvement in pathological alterations was observed (Fig. 4I).

**Fig. 5:**
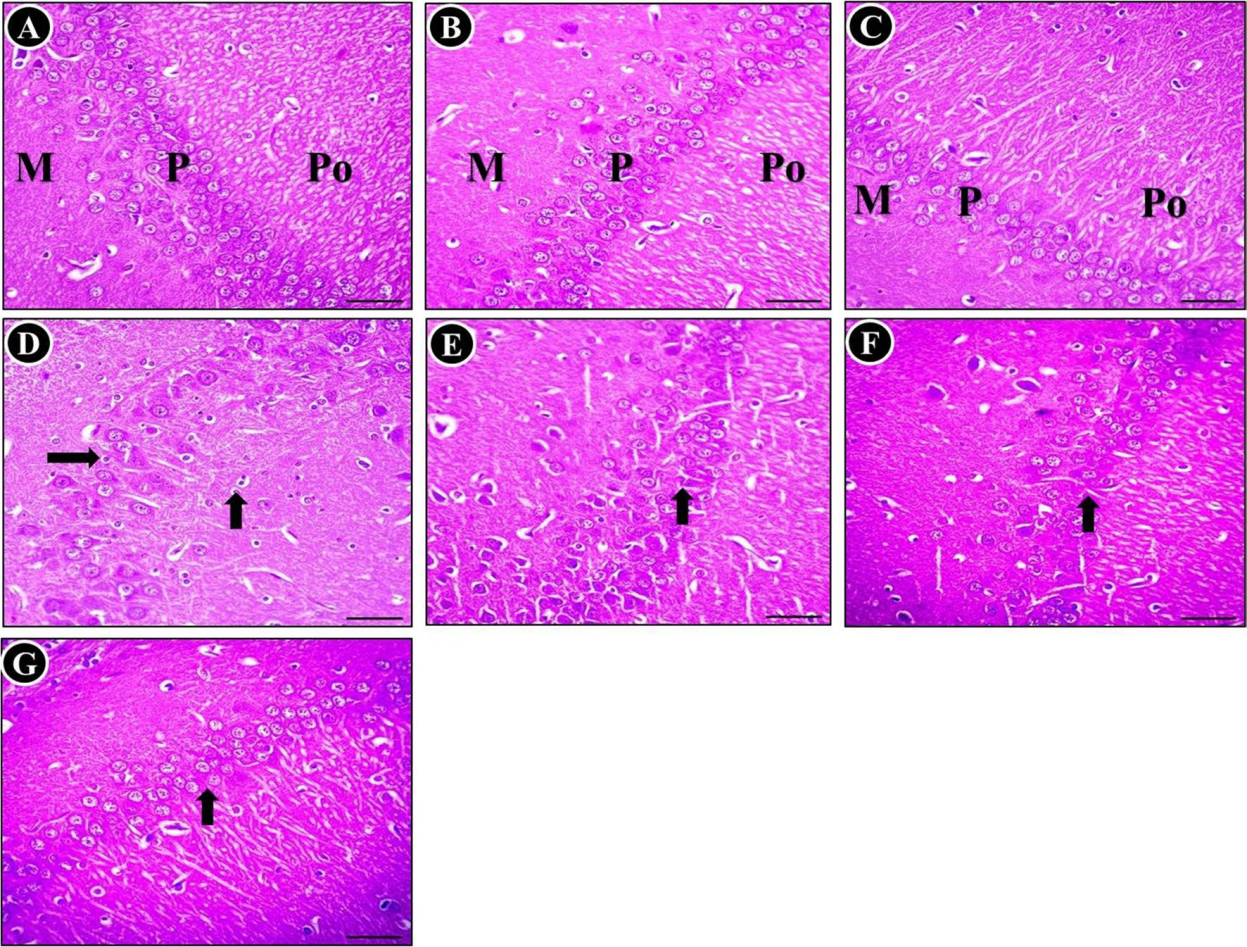
Histopathological changes in the hippocampus (H&E). **A**, **B**, and **C**. Normal hippocampal structure observed in the (control, SP, and TQ groups), comprising the molecular layer (M), pyramidal cell layer (P), and polymorphic layer (Po). Additionally, a few dark cells are discernible. **D**; MTX-treated group exhibiting pyramidal cell layer (black arrows) degeneration and reduced thickness (P), along with the presence of degenerated dark cells. In the SP + MTX-treated (**E**), TQ + MTX-treated (**F**), and SP + TQ + MTX combination (**G**) groups an amelioration of MTX-induced changes were observed

In the current investigation, the control groups exhibited a typical histological configuration of the hippocampus. This configuration consisted of three distinct layers, namely the molecular layer, the pyramidal cell layer characterized by a limited number of dark cells, and the polymorphic layer (Fig. 5A-C). Conversely, the group treated with MTX displayed significant alterations in the organization of these three layers, including disarray, absence of the close-knit arrangement of the granule neurons, and reduced thickness of the pyramidal cell layer. Additionally, the presence of degenerated dark cells was observed (Fig. 5D). In the groups where SP (Fig. 5E) or TQ (Fig. 5F) was administered concurrently with MTX intoxication, an amelioration of methotrexate-induced changes was observed. Furthermore, in rats treated with SP + TQ + MTX, a remarkable improvement in pathological alterations was observed (Fig. 5G). The histopathological lesion scoring of brain sections of the different experimental groups was recorded (Table 1).

**Table 1:** Histopathological Histopathological lesion scoring of brain sections of the control and MTX-exposed groups with and without SP and/or TQ treatments

Histopathological alteration	CON	SP	TQ	MTX	SP + MTX	TQ + MTX	SP + TQ + MTX
Degenerated neurons	-	-	-	+ + +	+ +	+	-
Dense eosinophilic cytoplasm	-	-	-	+ +	+	+	-
Chromatolysis	-	-	-	+	-	-	-
Pyknotic nucleus	-	-	-	+ + +	+ +	+ +	-
Shrunken degenerated Purkinje cells	-	-	-	+ + +	+ +	+	+
Loss of Purkinje cells	-	-	-	+ +	+	+	-
Reduced thickness of the pyramidal cell layer	-	-	-	+ +	+	+	+
Neuronal disarray	-	-	-	+	+	+	-

According to a modified semi-quantitative scale for the evaluation of histopathological changes, (-): none, ( +): mild, (+ +): moderate, (+ + +): severe grade

### Positive expression of Bax protein in rat cerebral, cerebellar, and hippocampal neurons

**Fig. 6:**
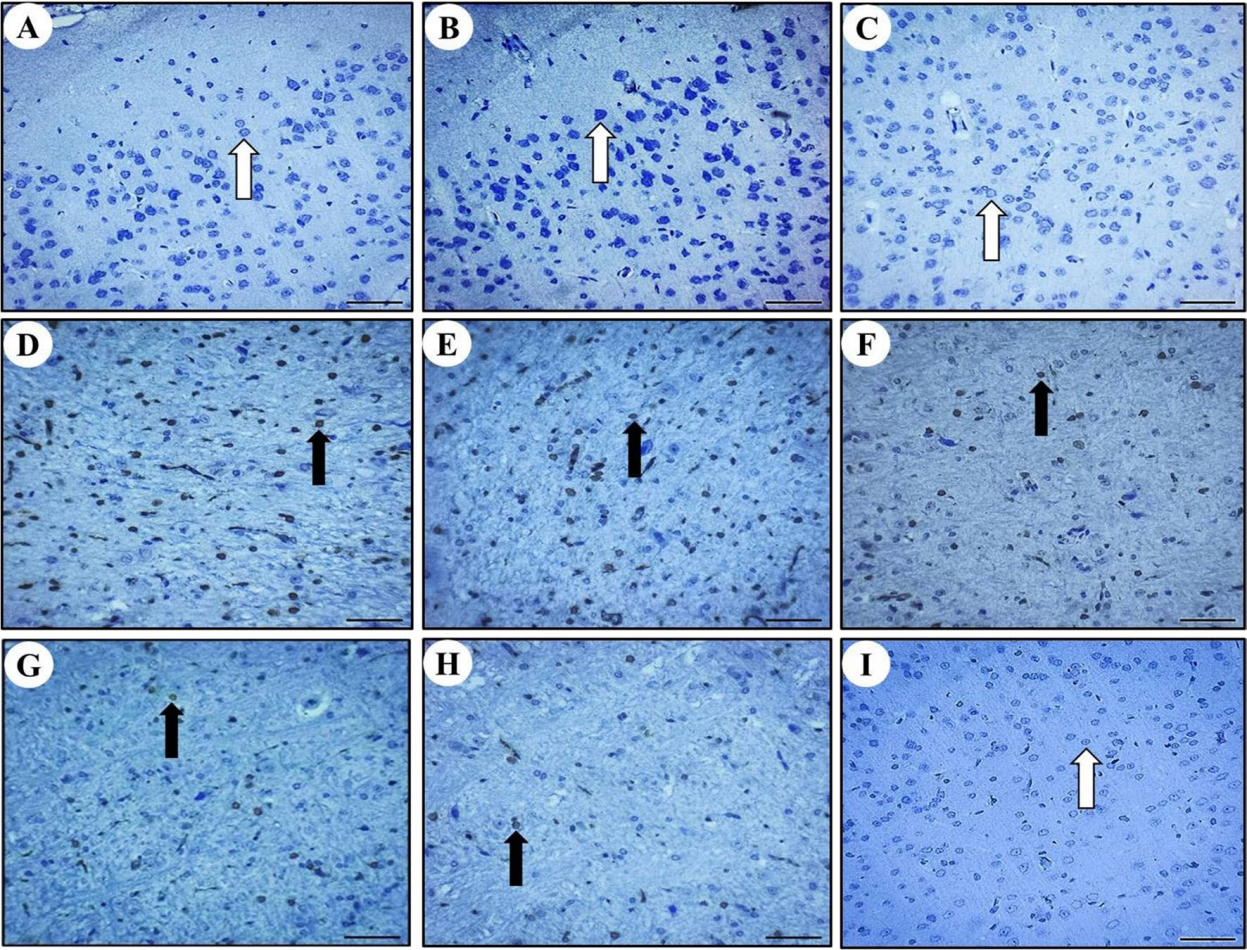
Photomicrographs of Bax immunohistochemical staining in the Cerebrum after MTX intoxication. **A** The normal control group shows BAX neurons. **B** SP group shows BAX negative neurons. **C** TQ group shows BAX negative neurons. **D**, **E**, and **F** MTX intoxicated group shows BAX-positive neurons. **G** SP + MTX treatment group, **H** TQ + MTX treatment group, and **I** SP + TQ + MTX treatment group. Black arrows show Bax positive neurons. White arrows indicate Bax -negative neurons

**Fig. 7:**
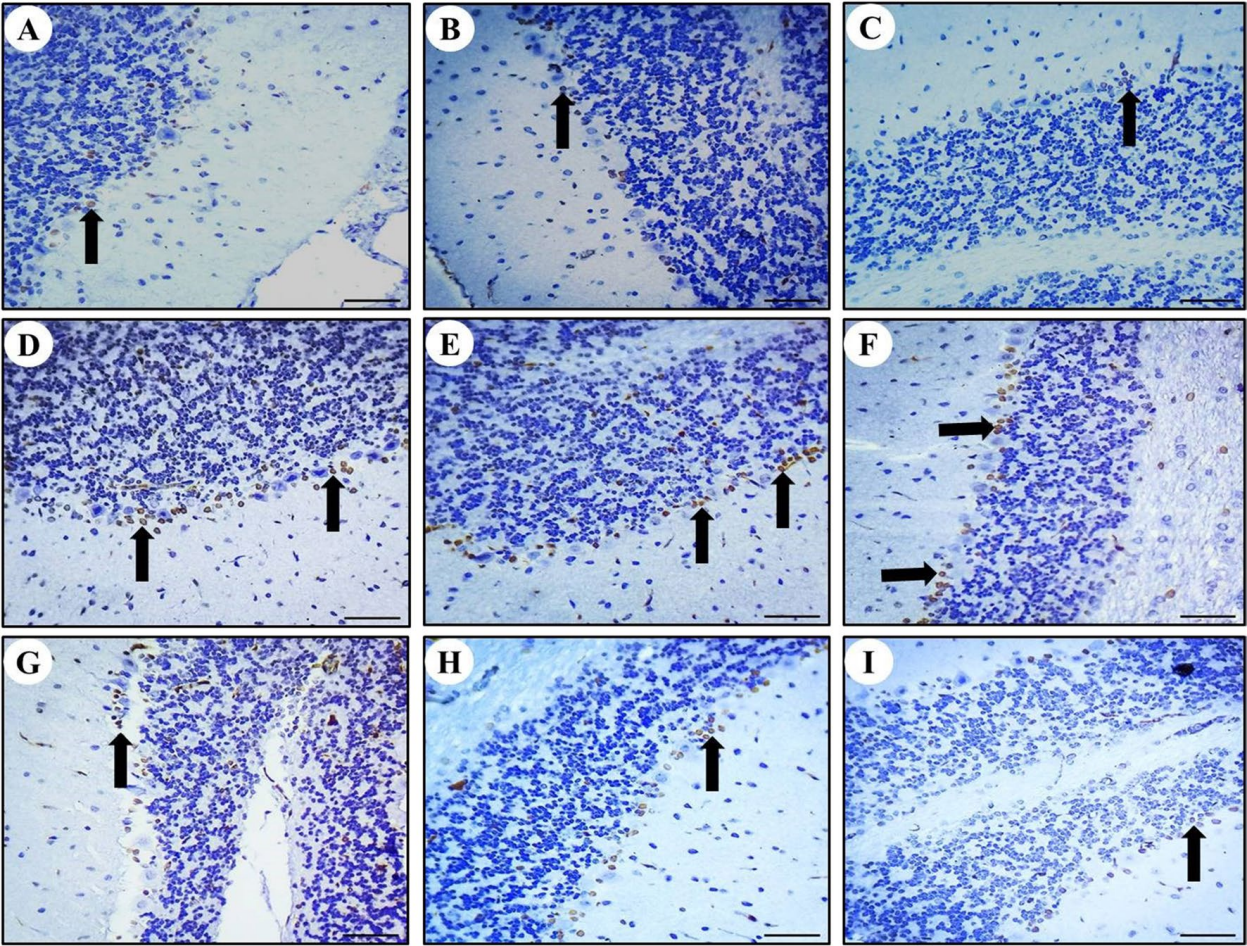
Immunohistochemical staining of Bax in rat cerebellum neurons. **A** The normal control group shows BAX neurons. **B** SP group BAX negative neurons. **C** TQ group shows BAX negative neurons. **D**, **E**, and **F** MTX intoxicated group shows BAX-positive neurons. **G** SP + MTX treatment group, **H** TQ + MTX treatment group, and **I** SP + TQ + MTX treatment group. Black arrows show Bax positive neurons. White arrows indicate Bax -negative neurons

**Fig. 8:**
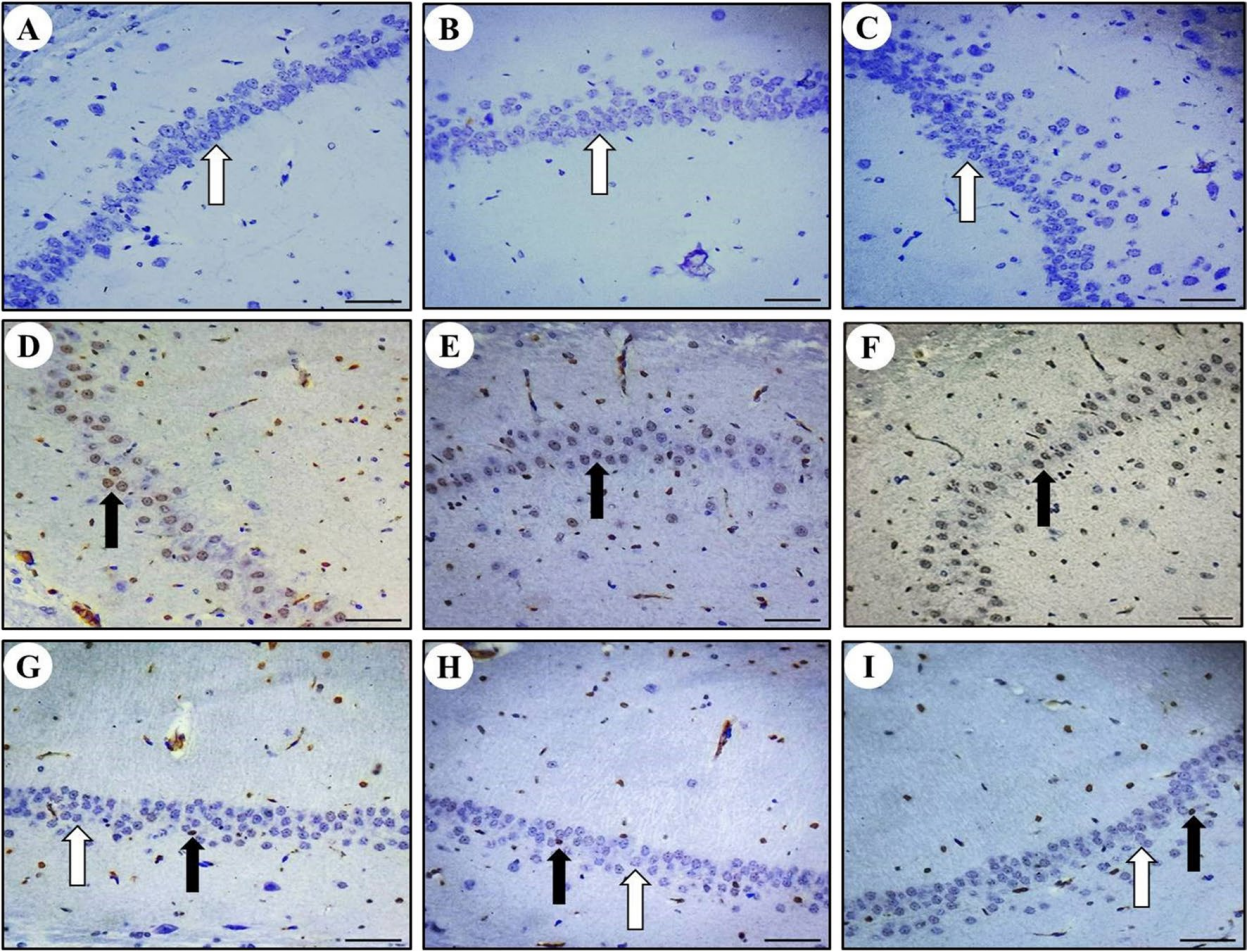
Immunohistochemistry staining of Bax in rat hippocampal region. **A** The normal control group shows BAX-negative neurons. **B** SP group BAX negative neurons. **C** TQ group BAX negative neurons. **D**, **E**, **F** The expression of Bax in the hippocampal region after MTX intoxication is shown by immunohistochemistry staining. **G** SP + MTX treatment group, **H** TQ + MTX treatment group, and **I** SP + TQ + MTX treatment group. Black arrows show Bax positive neurons. White arrows indicate Bax -negative neurons

**Fig. 9:**
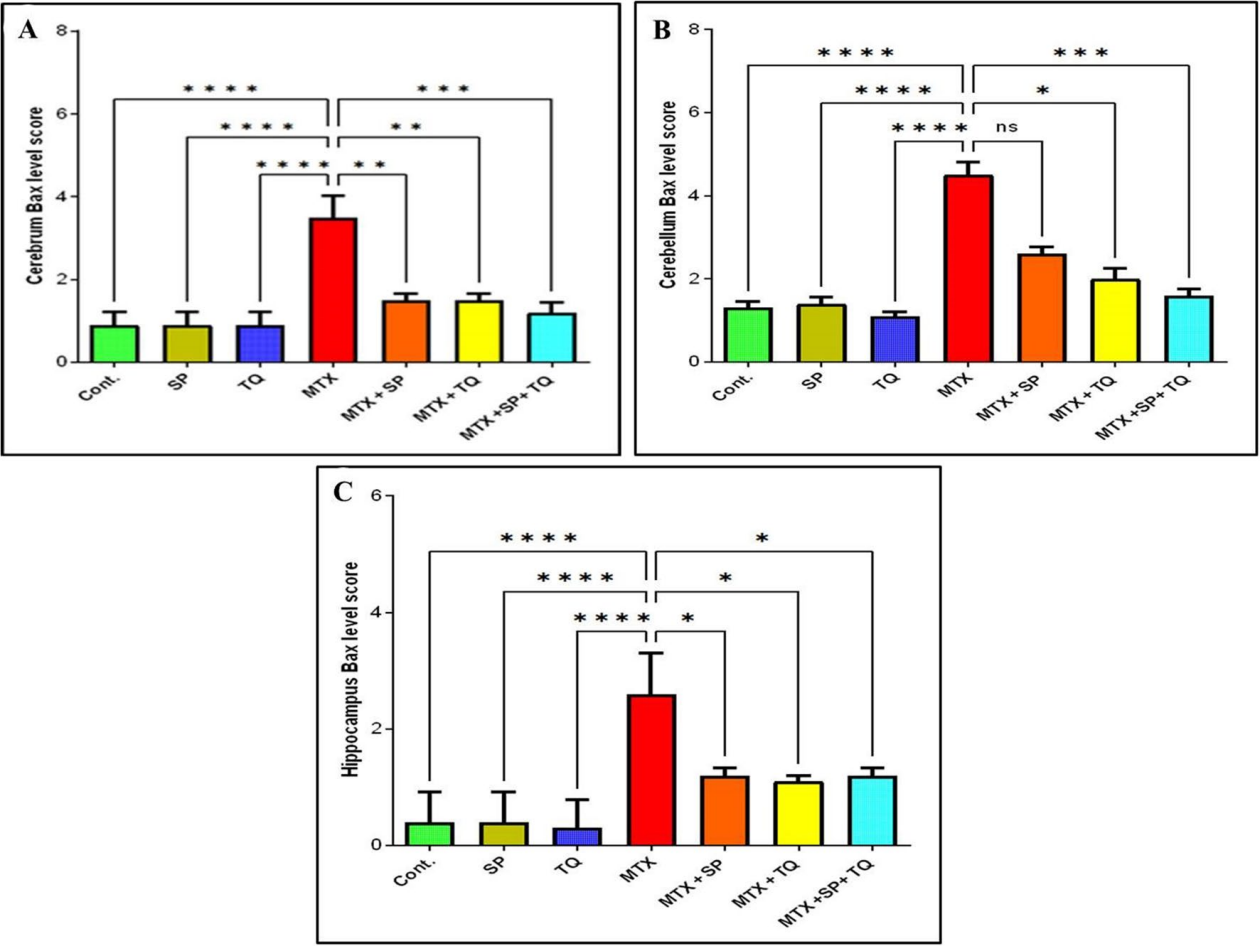
Scoring of Bax immunostaining intensity in the cerebrum, cerebellum, and hippocampal regions for comparative analysis among all experimental groups. **A** (cerebrum Bax level score); **B** (Cerebellum Bax level score); **C** (Hippocampus Bax level score). Statistical significance was determined by asterisks, with * indicating *p* < 0.05, ** indicating *p* < 0.01, *** indicating *p* < 0.001, **** indicating *p* < 0.0001, and ns; non-significant compared to the MTX-treated groups

The presence of Bax protein was observed as brown color particles primarily distributed in the cell membrane and cytoplasm in cerebrum (Fig. 6), cerebellum (Fig. 7), and hippocampus (Fig. 8). Compared to the control group, a significant increase in Bax-positive cells was observed in the MTX group's cerebral, cerebellar, and hippocampal neurons. However, in the SP + MTX, TQ + MTX, and SP + TQ + MTX treatment groups, the number of Bax-positive cells in brain neurons was significantly decreased compared to the MTX-intoxicated group. Scoring of Bax immunostaining intensity in the cerebrum, cerebellum, and hippocampal regions for comparative analysis among all experimental groups was illustrated (Fig. 9).

## Discussion

MTX is a commonly used chemotherapeutic agent, but it has negative effects on various organs, including the central nervous system (Aslankoc et al. 2022). Oxidative stress happens when the production of oxidants is greater than the body's ability to defend against them with antioxidants. This imbalance can result in oxidative damage to lipids, proteins, and DNA in cells (Aboubakr et al. 2023b). Research has shown that oxidative stress is a primary cause of organ damage in experimental studies (Daggulli et al. 2014). MTX can cause an increase in oxidative stress in various organs, including the brain. White matter is particularly susceptible to oxidative damage due to its high concentration of polyunsaturated fatty acids and low levels of antioxidants (Famurewa et al. 2017). This leads to an increase in lipid peroxidation induced by MTX, which results in the degradation of membrane permeability, neurotransmitter-receptor interaction, and cell apoptosis in the cerebral cortex (Welbat et al. 2020). Studies have shown that ROS generated by MTX can deplete antioxidant activity in the rat cerebral cortex under oxidative stress (Famurewa et al. 2019).

The generation of superoxide radicals is an outcome of oxidative stress. These radicals transform into hydrogen peroxide (H_2_O_2_), which can travel to various tissues and organs away from its origin and penetrate the cell membrane. The presence of transitional metals in the area where H_2_O_2_ arrives generates hydroxyl radicals, which are more harmful than superoxide radicals. This leads to further elevation of oxidative stress, causing neurotoxicity (Welbat et al. 2020; Ahmed et al. 2021).

Numerous studies have highlighted the role of oxidative stress in neurodegenerative diseases (Kim et al. 2015; Liu et al. 2017; Suwannakot et al. 2022). The present investigation demonstrated that MTX treatment resulted in a significant reduction in the activities of SOD and CAT enzymes, as well as a decrease in the concentration of GSH in brain tissue. MDA is widely recognized as the end product of lipid peroxidation and is commonly employed as a reliable marker for measuring oxidative stress (Ayala et al. 2014). Therefore, an increase in the accumulation of ROS induced lipid peroxidation leads to a breakdown in the cell membrane, resulting in alterations of protein structure and function, and DNA damage (Su et al. 2019; Juan et al. 2021). Previous studies have demonstrated that MTX administration results in elevated levels of MDA and decreased levels of antioxidant enzymes in the brain (Tongjaroenbuangam et al. 2013; Famurewa et al. 2019; Shalaby et al. 2019; Welbat et al. 2020; Ahmed et al. 2021; Sritawan et al. 2021). The current study revealed that rats treated with MTX had increased levels of oxidative stress, as evidenced by decreased levels of SOD, CAT, and GSH in brain tissue. However, co-administration of SP or TQ with MTX resulted in a decrease in MDA levels. SP or TQ possess antioxidant properties and can act as free radical scavengers to inhibit the formation of ROS. These substances also have the ability to reduce oxidative stress by decreasing MDA levels and activating enzymatic antioxidants, such as SOD, CAT, and GSH (Aboubakr et al. 2021; Bin-Jumah et al. 2021).

In our study, we observed a significant increase in serum AchE activity after MTX administration, which is an important marker for detecting neurotoxicity. The elevated levels of AchE may be an indicator of MTX-induced neurotoxicity in the brain (Verma et al. 2018). Co-administration of SP or TQ with MTX increased the levels of AchE, which is consistent with the findings of other studies that have reported the protective effects of SP against lead-induced toxicity (Galal et al. 2019) and TQ against chlorpyrifos-induced toxicity (Aboubakr et al. 2021).

Excessive production of ROS and oxidative stress have been shown to trigger intracellular cascade signaling that up-regulates the expression of proinflammatory genes and promotes the release of inflammatory cytokines (Aboubakr et al. 2021). Consistent with this, the current study found that MTX-induced oxidative stress in the brain tissues resulted in increased levels of IL-1β, IL-6, and TNF-α in the serum.

Previous clinical and experimental research has highlighted that macrophages and monocytes secrete cytokines in response to tissue damage caused by harmful events (Asvadi et al. 2011). The process of inflammation in biological systems is closely linked to the production of ROS, oxidative stress, and cellular damage in different types of cells (Chumphukam et al. 2018). Moreover, previous research studies have established a connection between MTX toxicity and inflammation (Yang et al. 2012; Kandemir et al. 2017). The present study demonstrates that injection of MTX induces neuroinflammatory responses as evidenced by significant increases in cerebral levels of TNF-α, IL-1β, and IL-6, and histology showing necrosis and inflammatory cells infiltration. Activation of transcription factors associated with inflammatory responses is implicated in pro-inflammation caused by oxidative stress induced by MTX (Famurewa et al. 2019). Oxidative stress induced by MTX may act as a signaling mechanism for the recruitment of inflammatory cells like macrophages, neutrophils, and leukocytes, which are involved in cytokine production (Chumphukam et al. 2018). Our findings are consistent with previous studies that have reported MTX-induced pro-inflammation in the sciatic nerve and hepatorenal system, which is characterized by markedly increased levels of TNF-α, IL-1β, and IL-6 (Kandemir et al. 2017; Khafaga and El-Sayed 2018). The levels of inflammatory mediators in the cerebrum were significantly reduced by oral administration of SP or TQ in this study. SP has been shown to have anti-inflammatory properties in previous studies by (Okuyama et al. 2017), and has been effective against lead-induced neurotoxicity (Khalil et al. 2018), acrylamide (Bin-Jumah et al. 2021), and microcystin-LR (Germoush et al. 2022). TQ has been shown to have anti-inflammatory and antioxidant properties by scavenging ROS and upregulating antioxidants in formaldehyde (Saygin et al. 2018), microcystin-LR (Abdel-Daim et al. 2019), acrylamide (Abdel-Daim et al. 2020), and chlorpyrifos (Aboubakr et al. 2021) induced toxicity. The oxidative damage and pro-inflammatory cytokines observed in this study could increase the susceptibility of the brain to various neurodegenerative diseases.

The MTX group exhibited significant histopathological changes, such as vacuolar alterations, apoptotic cells, and infiltration of inflammatory cells, which have been reported in previous studies (Famurewa et al. 2019; Shalaby et al. 2019; Ahmed et al. 2021; Aslankoc et al. 2022). However, administration of SP or TQ decreased the neurological lesions, as demonstrated in studies on neurotoxicity induced by lead, nicotine, doxorubicin, and formaldehyde (Khalil et al. 2018; Saygin et al. 2018; Galal et al. 2019; Elsonbaty and Ismail 2020).

Bax, a protein that promotes cell death (apoptosis), undergoes translocation to the mitochondria during the early stages of apoptosis, indicating its crucial role in transmitting signals related to cell death (Sritawan et al. 2023). When brain tissues are exposed to MTX, it leads to an increase in the expression of Bax. MTX induces apoptosis by generating reactive oxygen species (ROS), which in turn causes oxidative stress (Ali et al. 2014). Previous studies observed an upregulation of pro-apoptotic Bax expression in rats treated with MTX (Ashok and Sheeladevi 2014; Sritawan et al. 2023). Conversely, when TQ was administered together with ACR, the expression of Bax decreased, as previously reported by (Hosseinzadeh et al. 2007; Tabeshpour et al. 2020). This decrease in Bax expression suggests that the neuroprotective mechanism of TQ may involve its anti-apoptotic activity. In a study by (Khalil et al. 2018), it was suggested that spirulina could be an effective therapeutic agent that enhances cell survival in rat brains by suppressing cell death.

The beneficial effects of SP may be attributed to its abundance of antioxidants such as C-phycocyanin, β carotene, lipids, proteins, essential (amino acids & fatty acids), vitamins carbohydrates and minerals, all of which possess strong anti-inflammatory and antioxidant properties (Germoush et al. 2022).

According to the findings of this research, TQ has the potential to normalize the levels of inflammatory cytokines and biomarkers of neuronal injury in the serum, while also reducing oxidative stress and peroxidation of lipids in the brain tissue. Thymoquinone, the primary component of Nigella sativa, has been shown to possess antioxidative properties in previous research studies (Hosseinzadeh et al. 2007). Thymoquinone, when in its reduced form (thymohydroquinone), serves as an electron donor that counteracts hydroxyl and superoxide radicals by preventing their attack on polyunsaturated fatty acids in cell membranes. This strong capacity to scavenge free radicals can explain the potent antioxidative effects of TQ (Khither et al. 2018). Additionally, the present study found that TQ reduced inflammatory biomarkers in the serum, either directly through the suppression of their expression levels or by mitigating oxidative stress (Firdaus et al. 2018). Comparable outcomes have also been documented for TQ in its ability to combat neurotoxicity caused by acrylamide (Abdel-Daim et al. 2020).

## Conclusions

The study revealed that MTX exposure could have detrimental effects on brain markers in rats. The neurodegenerative potential of MTX is attributed to oxidative stress, inflammation, and disrupted neurotransmission. However, SP and TQ supplements were found to exert neuroprotective effects against MTX-induced neuronal injury by utilizing their antioxidant, anti-inflammatory, and neuromodulatory potentials. Therefore, this study suggests that SP or TQ could be used as potential pharmacological treatments for cancer patients exposed to MTX.

## Data Availability

The data presented in this study are available upon request from the first author.
